# Insights on the Quantitative Concurrent Fluorescence-Based Analysis of Anti-COVID-19 Drugs Remdesivir and Favipiravir

**DOI:** 10.1007/s10895-022-02998-z

**Published:** 2022-06-30

**Authors:** Mohamed El-Awady, Heba Elmansi, Fathalla Belal, Rasha abo Shabana

**Affiliations:** 1 Pharmaceutical Analytical Chemistry Department, Faculty of Pharmacy, Mansoura University, Mansoura, 35516 Egypt; 2 Department of Pharmaceutical Chemistry, Faculty of Pharmacy, Delta University for Science and Technology, International Coastal Road, Gamasa 11152, Mansoura, Egypt

**Keywords:** Remdesivir, Favipiravir, Spectrofluorometric, Synchronous, Plasma

## Abstract

We hereby introduce a sensitive fast straightforward spectrofluorometric method for the estimation of remdesivir and favipiravir. The two drugs are prescribed in some regimens to treat COVID‐19 pandemic disease, which is caused by SARS‐CoV‐2. The method is based on the first derivative synchronous spectrofluorimetry approach for the measurement of remdesivir and favipiravir. This was accomplished at 251 nm and 335 nm respectively using the first derivative order at delta lambda of 140 nm. A linear response with a correlation coefficient 0.9994 was achieved between the concentration and the derivative amplitudes in the ranges of 20.0–100.0 ng ml^−1^ and 40.0–100.0 ng ml^−1^ for remdesivir and favipiravir, respectively. The methods were validated for different parameters as stated by the pharmacopeial rules and were applied successfully for estimation of the studied drugs in their synthetic mixtures and in spiked human plasma samples. No significant difference was observed between the proposed and comparison methods as revealed from the analysis of data.

## Introduction

COVID-19 is a disease that has made severe disturbance to humanity across the world. This disease initiated by SARS‐CoV‐2 is a single‐stranded RNA virus that has high transmission rate and infectivity compared to other viruses. Researchers focused on the treatment development and controlling measures against coronavirus. Different antiviral therapies have exhibited satisfying results from which, remdesivir and favipiravir are two such drugs. It was found that these drugs inhibit viral enzyme RNA-dependent polymerase and thereby have therapeutic potential in the treatment of COVID-19 [1].

**Fig. 1:**
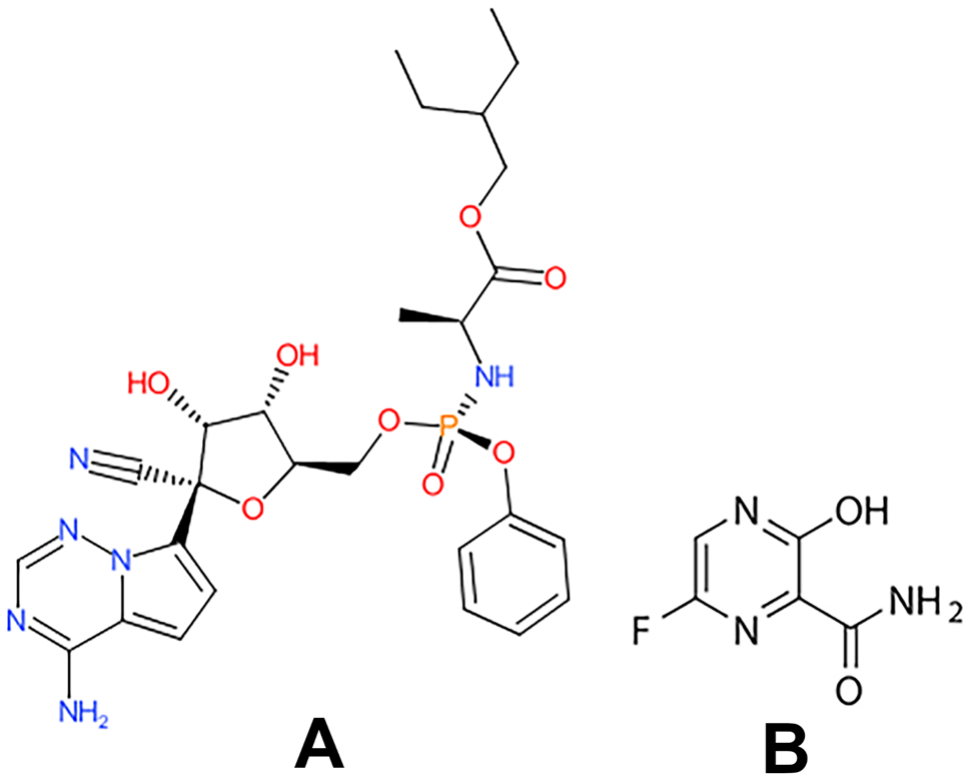
Structural formulae of (**A**) RMD, (**B**) FVP

Remdesivir (RMD), as in Fig. 1A: is 2-ethylbutyl (2*S*)-2-[[[(2*R*,3*S*,4*R*,5*R*)-5-(4-aminopyrrolo[2,1-f][1,2,4]triazin-7-yl)-5-cyano-3,4-dihydroxyoxolan-2-yl]methoxy-phenoxyphosphoryl]amino]propanoate [2]. It inhibits the viral RNA-dependent, RNA polymerase with in vitro inhibitory activity against SARS-CoV-1 [3]. It is observed that treatment with RMD may prevent the progression to more severe respiratory disease [4].

Favipiravir (FVP), as in Fig. 1B; is 5-fluoro-2-oxo-1*H*-pyrazine-3-carboxamide [5]. It is a purine nucleic acid analog and one of the drugs that are indicated for the treatment of patients with mild to moderate COVID-19 disease [6]. It is a pyrazine carboxamide derivative, having antiviral activity against a number of RNA viruses [7]. FVP was first introduced by Toyama Japanese Company. Later, it was permitted in Japan for influenza treatment [8, 9].

Several trials have been started to assess the safety and efficiency of RMD and FVP in COVID‐19 patients [10, 11]. Latest findings recommend RMD and FPV as antiviral agents for short term combating of COVID‐19 [6].

The literature discusses various methods for the determination of the two antiviral drugs RMD and FVP either separately or with other drugs including spectroscopy [12, 13], HPLC [12–18], electrochemistry [19, 20], spectrofluorimetry [18, 21] and capillary electrophoresis [22]. A review that covers most of the analytical methods developed for the quantitative determination of RMD in biological matrices is recently published [23]. In the previous reported methods; the low sensitivity of spectrophotometric methods was an obvious disadvantage [12, 13]. From the economic and environmental aspects, liquid chromatography is not favored because of extensive volumes of highly pure organic solvents and tedious sample preparation procedures. Additionally, HPLC approach is often very costly, and this expense can be prohibitive for clinical laboratories. As a result, we aimed to provide another cost effective alternative. Till present, no spectrofluorometric method was yet reported for the concurrent determination of RMD and FVP together although the two drugs are reported to exhibit native fluorescence [18, 21].

In this study, a new method is proposed for the simultaneous estimation of the two antiviral remedies at the nanogram level. The accessibility of the spectrofluorometric technique is beneficial in quality control analyses and in laboratories that lack costive or complicated operating systems. This work describes the application of the new methodology for the quantification of the binary mixture of RMD and FVP in different laboratory-prepared mixtures without prior separation. Moreover, the ease and sensitivity of the method allow quantitative measuring of the drug in samples of human plasma.

## Experimental

- Instrumentation, chemicals and materials

All the measurements were recorded using Cary Eclipse fluorescence spectrophotometer (Agilent Technologies), equipped with a xenon lamp and the spectra were smoothed with a factor of 20. The data were manipulated by Cary Eclipse software to calculate the first order of the synchronous spectra of the drugs. Data were obtained using delta lambda of 140 nm at 251 nm and 335 nm for RMD and FVP, respectively.

Samples of pure RMD and FVP were kindly donated by EIPICo, Egypt. Solvents (HPLC grade) were bought from Sigma‐Aldrich (Germany). Chemicals for the preparation of different buffers were purchased from El‐Nasr Pharmaceutical Chemicals Co., Egypt. Analytical grade chemicals were used throughout the work.

Human plasma samples were obtained from the Egyptian National Blood Bank, Mansoura, Egypt, and kept frozen at -20 °C until use, then gentle thawing is performed.

## Sample Preparation

- Preparation of standard and working solutions:

A 100.0 μg/mL standard ethanolic solution of RMD and FVP were prepared in separate 100 mL volumetric flasks by dissolving 10 mg of each drug in ethanol. Then we completed the volume to the mark with the same solvent. Working standard solutions were then prepared by subsequent dilution with ethanol.

- Preparation of biological samples:

Plasma samples were kept at kept frozen at -20 °C, then subjected to gentle thawing before use. 1 mL from the samples were transferred in centrifugation tubes to procced in the method development.

## Method Development and Applications

1. For calibration curves; different concentrations from both RMD and FVP working solutions in the range of 20.0–100.0 ng mL^−1^ and 40.0–100.0 ng mL^−1^, respectively were transferred into 10-mL volumetric flasks and completed with ethanol. Each drug was measured separately using synchronous fluorometry at Δλ = 140 nm, then the spectra were converted to the first derivative order using Cary Eclipse software. Finally, RMD and FVP were measured at 251 nm and 335 nm respectively to construct calibration curves. The corresponding regression equations were then derived.
2. For investigation of synthetic mixtures of both drugs; different concentrations were prepared together in 10-mL measuring flasks in ethanol to reach the following concentrations: (100.0,30.0), (50.0,50.0), (40.0,80.0), (30.0,100.0) ng mL^−1^. The same procedure for calibration curves was followed to calculate percentage recoveries.
3. For spiked plasma samples, aliquots (1.0 mL) of plasma samples were transferred into centrifugation tubes. The samples were spiked with different concentrations of each drug in ascending manner to locate the final concentrations within the linear range (20.0–100.0 ng mL^−1^ and 40.0–100.0 ng mL^−1^) for RMD and FVP, respectively. These tubes were mixed well, and acetonitrile was added. The final volume was adjusted to be 5.0 mL. Samples were vortex mixed for 3 min, then centrifuged at 3000 rpm for 30 min. 1.0 mL aliquots of the upper layers were quantitatively transferred into another set of 10 mL volumetric flasks and a blank experiment was carried out concurrently. The general procedures described for the calibration curves were followed. Specific calibration curves were constructed for each drug and mixtures of two drugs were also investigated inside the biological matrix.

## Results and Discussion

**Fig. 2:**
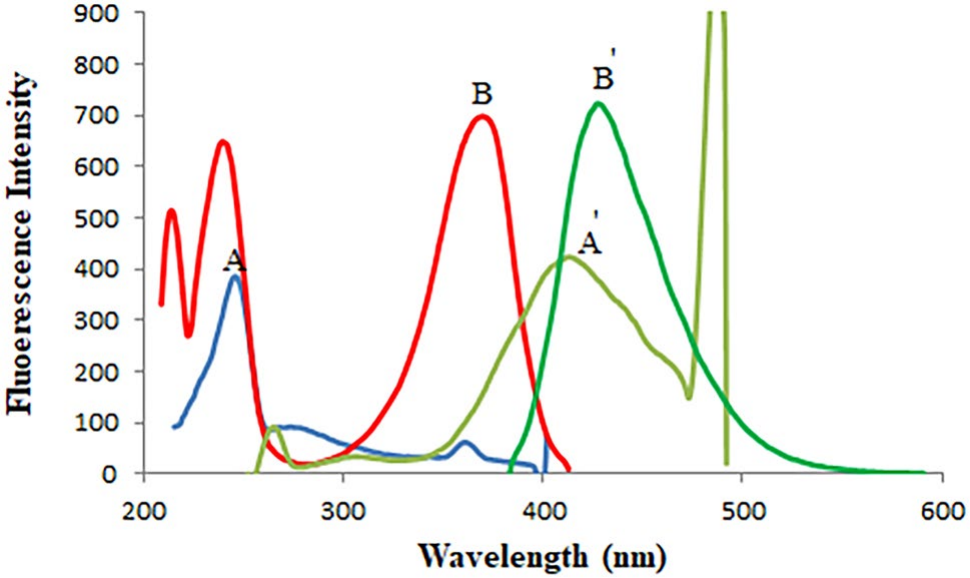
Excitation and emission fluorescence spectra of RMD (30 ng/mL) (**A**, **A'**), FVP 20 ng/mL (**B**, **B'**) in methanol

**Fig. 3:**
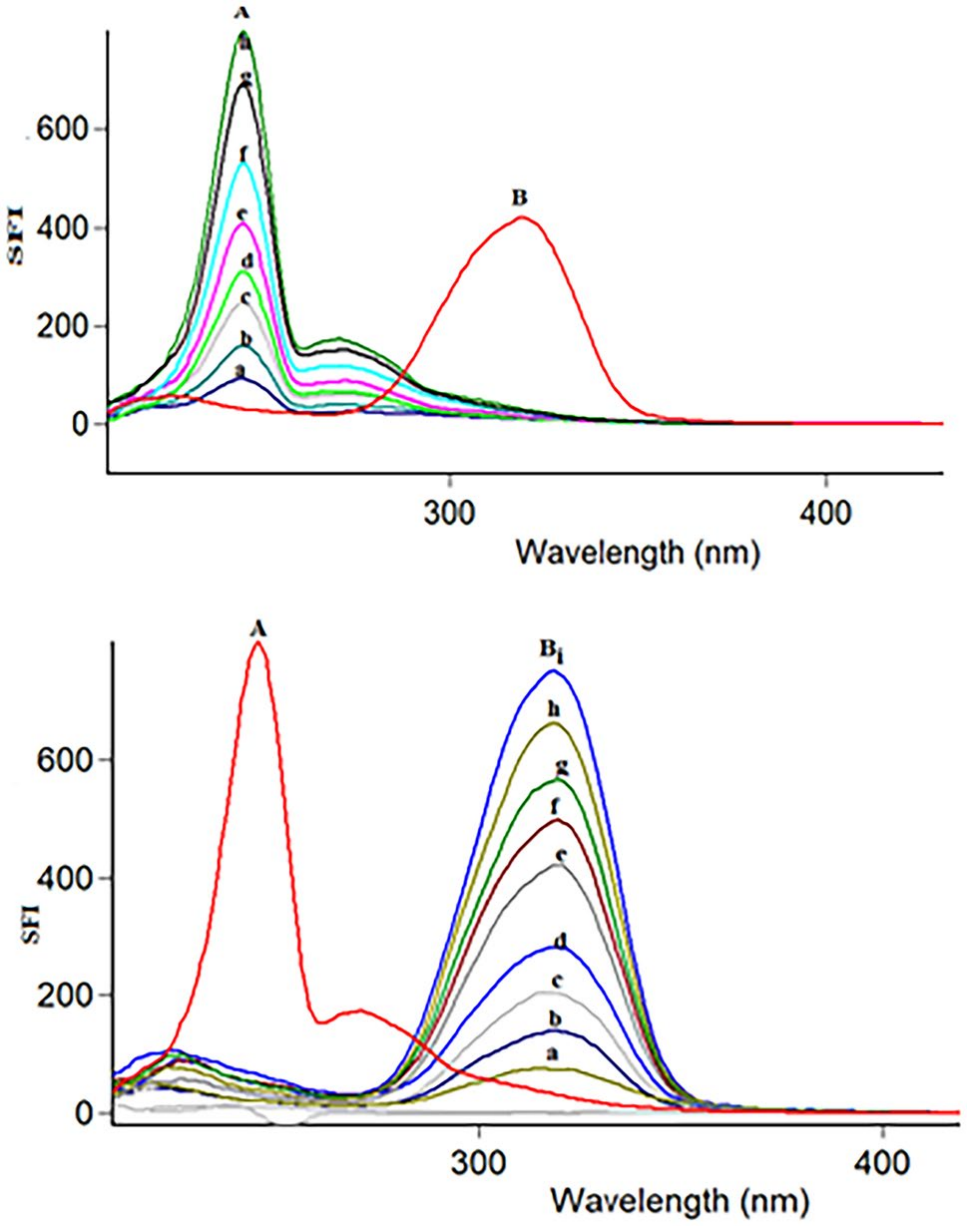
A-Synchronous fluorescence spectra of: i-(**A**) **a–h**, RMD (10.0–100.0 ng/mL). (**B**) FVP (60 ng/mL). ii- Synchronous fluorescence spectra of: (**A**) RMD (100 ng/mL) (**B**) **a**–**i**, FVP (20–100 ng/mL)

**Fig. 4:**
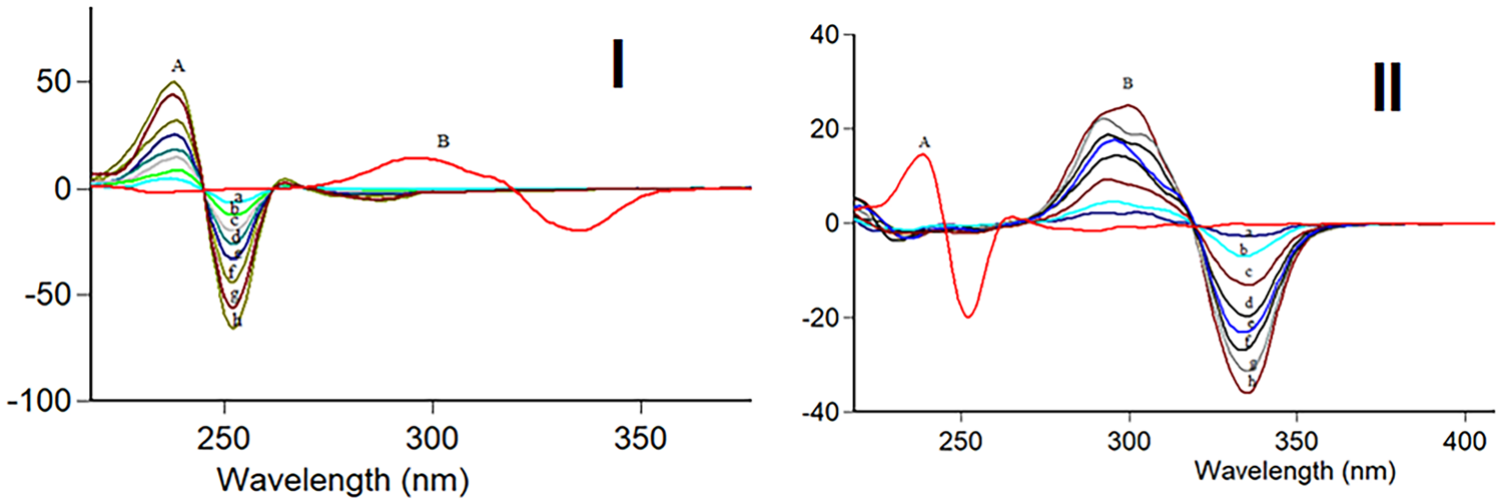
First derivative synchronous fluorescence spectra of: i- (**A**) (**a**–**f**) of RMD (10.0–100.0 ng/mL) at 251 nm (**B**) FVP (60.0 ng/mL) ii- (**A**) RMD (60.0 ng/mL) (**B**) (**a**–**f**) of FVP (20.0–100.0 ng/mL) at 335 nm

Fluorescence is the emission of light from any substance and occurs from electronically excited states. Overlapping spectrum is a common problem in resolving more than one drug (Fig. 2). The satisfactory resolution of mixtures always can be performed by synchronous spectrofluorimetry [24]. It is also called Stokes shift emission spectroscopy [25]. In such technique, the signal is recorded by simultaneously scanning the excitation and emission wavelengths at the same speed with a fixed wavelength (Δλ) between the excitation and emission wavelengths. This method uses an inexpensive solvent, and relatively the utilized instrument is commonly available in most quality control laboratories. In our study, minor overlap still occurs in synchronous spectrofluorimetry as illustrated in Fig. 3. Therefore, we aimed to estimate RMD and FVP simultaneously based on a sensitive first derivative synchronous method to remove any interference and increase the selectivity of the method (Fig. 4).

The parameters associated with the sensitivity, repeatability, and accuracy of the method were evaluated individually including solvent, pH, surfactants, and Δλ.

**Fig. 5:**
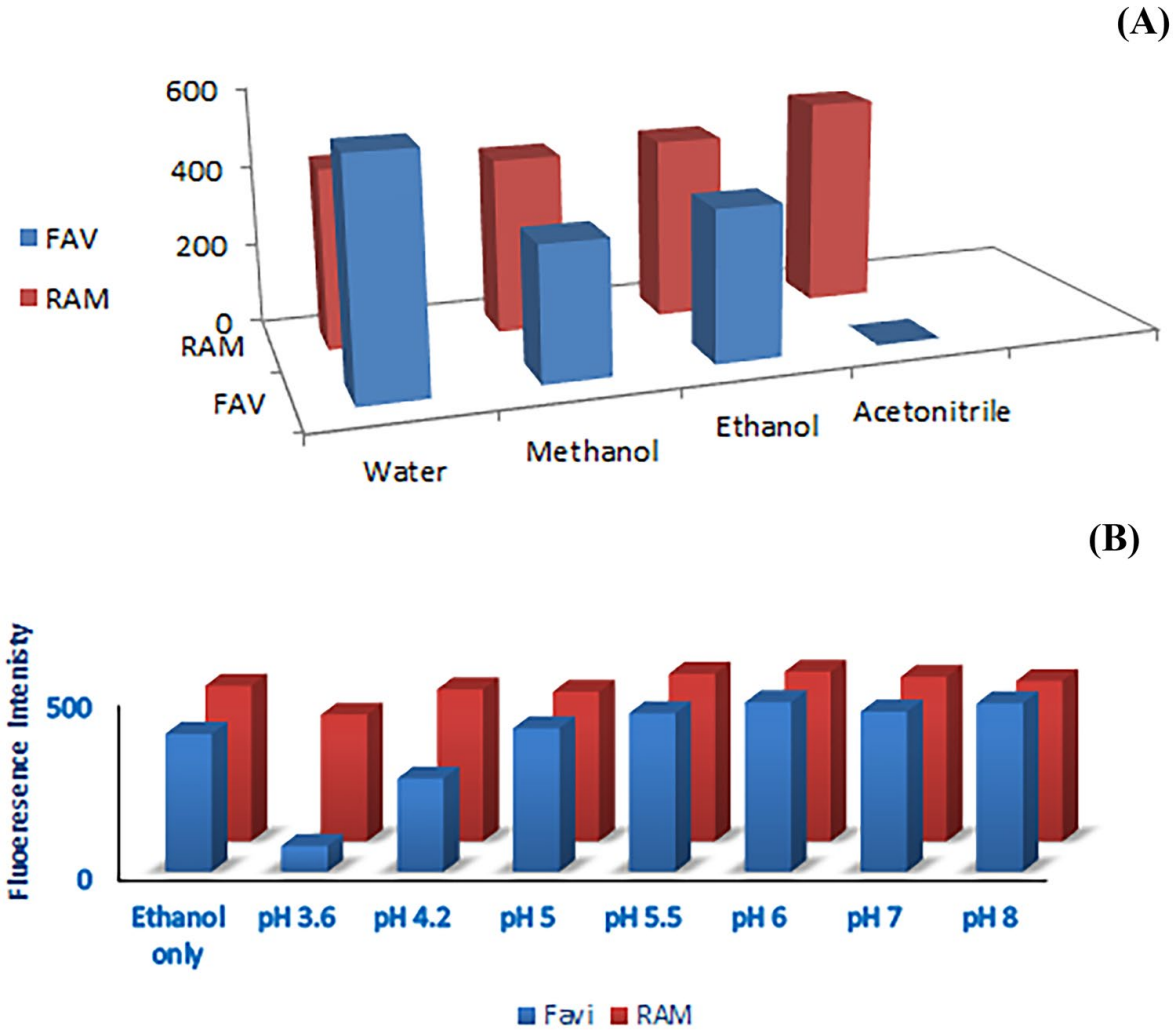
**A** Effect of diluting solvent on relative synchronous fluorescence intensity for RMD and FVP **B** Effect of different pH on relative synchronous fluorescence intensity for RMD and FVP

Solvents may have a significant effect in synchronous fluorometry, as they may influence resolution of spectra, blank and sensitivity. Water, ethanol, methanol, and acetonitrile were tried. Water resulted in the highest sensitivity; however, it affects resolution of RMD and FVP. Hence, ethanol was chosen in this study to compromise separation and sensitivity as shown in Fig. 5.

Different pH values were investigated using different buffers, as illustrated in Fig. 5 using Britton Robinson and borate buffers from 3.6 to 8. The optimum condition was attained using ethanol only without adding any buffer.

Surfactants were studied to reach maximum sensitivity using micellar media above critical micelle concentration. Anionic surfactant (SDS), nonionic surfactant (Tween-80) and (cremophor RH-40), cationic surfactant (cetrimide) were tried using 1 mL of each surfactant (1.0% w/v). No significant enhancement was achieved using any of these surfactants.

The selection of optimum Δ λ is an important factor as it could significantly affect the resolution, sensitivity and spectra symmetry. Thus, variable Δ λ values (20–160 nm) was carefully investigated. Δ λ of 140 nm revealed the best band shapes with the highest sensitivity for both drugs.

## Validation Parameters

Under the optimized conditions, a linear response was obtained between the first derivative amplitudes and the concentrations for each of RMD and FVP over the ranges of 20.0–100.0 ng ml^−1^ and 40.0–100.0 ng ml^−1^, respectively with the following regression equations:

$$\mathrm{Y}=0.067X-0.093\;\mathrm{for}\;\mathrm{RMD}$$

$$\mathrm{Y}=0.415X-5.620\;\mathrm{for}\;\mathrm{FVP}$$

where Y is the first derivative amplitude and X is the corresponding concentration.

Limits of quantitation and detection were computed mathematically following ICH guidelines [26]. LOQ values were found to be 8.57, 10.79 ng/ mL and LOD were 2.83, 3.62 ng/mL for RMD and FVP respectively. Table 1 summarizes the validation data for the designated methodology.

**Table 1:** Validation parameters according to ICH guidelines

Parameters	RMD	FVP
Linearity range (ng/mL)	10.0–100.0	20.0–100.0
Intercept (*a*)	-0.093	-5.620
Slope (*b*)	0.067	0.415
Correlation coefficient (*r*)	0.9994	0.9994
S.D. of residuals (S_*y/x*_)	0.811	0.477
S.D. of intercept (S_*a*_)	0.577	0.455
S.D. of slope (S_*b*_)	0.010	0.006
Percentage relative standard deviation, % RSD	1.79	1.56
Percentage relative error, *%* Error	0.68	0.59
Limit of detection, LOD (ng/mL)	2.83	3.62
Limit of quantitation, LOQ (ng/mL)	8.57	10.97

Accuracy has been determined by calculating mean percent recoveries of seven concentration for each drug and comparing the results with previous reports [12, 17]. No significant difference was found regarding accuracy and precision, respectively as revealed in Table 2 [27].

**Table 2:** Application of the proposed method for the assessment of RMD and FVP in pure forms

Studied drugs	Amount taken (ng/mL)	Amount found (ng/mL)	% Found	Comparison methods [12, 17] % Found
RMD	10.0	10.062	100.62	98.74
	20.0	19.566	97.83	101.12
	30.0	29.355	97.85	99.64
	40.0	40.038	100.10	
	60.0	60.953	101.59	
	80.0	81.720	102.15	
	100.0	98.327	98.33	
$\overline x$ ± S.D			99.78 ± 1.79	99.83 ± 1.20
*t*			0.045 (2.30)	
*F*			2.23 (19.32)	
FVP	20.0	20.126	100.63	98.9
	30.0	30.241	100.8	101.16
	60.0	61.21	102.02	99.61
	70.0	69.423	99.18	
	80.0	78.536	98.17	
	90.0	88.733	98.59	
	100.0	101.952	101.95	
$\overline x$ ± S.D			100.19 ± 1.56	99.89 ± 1.16
t*			0.38 (2.30)	
F*			1.83 (19.32)	

***N.B.*** Each value is the mean of three separate determinations

^*^ The tabulated t and F values are respectively at *p* = 0.05 [27]

The precision of the method was investigated (as RSD %) through assessing intra-day precision and inter-day precision over three levels of concentrations (20.0,40.0,80.0 ng/mL for RMD) and (30.0,60.0,90.0 ng/mL for FVP). The results of this assay are summarized in Table 3.

**Table 3:** Precision data for the assessment of RMD and FVP by the proposed method

Amount taken (ng/mL)	% Found	% RSD	% Error
**RMD**			
Intraday 20.0	98.58 ± 0.57	0.58	0.33
40.0	100.6 ± 0.94	0.93	0.54
80.0	100.92 ± 1.87	1.85	1.07
Interday 20.0	98.93 ± 1.04	1.05	0.60
40.0	100.43 ± 0.60	0.60	0.34
80.0	101.47 ± 0.55	0.54	0.31
**FVP**			
Intraday 30.0	100.61 ± 1.24	1.23	0.711
60.0	101.05 ± 1.00	0.99	0.57
90.0	99.00 ± 0.63	0.64	0.57
Interday 30.0	100.10 ± 1.44	1.43	0.83
6.0	100.37 ± 1.56	1.56	0.89
90.0	99.37 ± 0.44	0.44	0.26

## Application in Different Matrices and Selectivity Evaluation

**Fig. 6:**
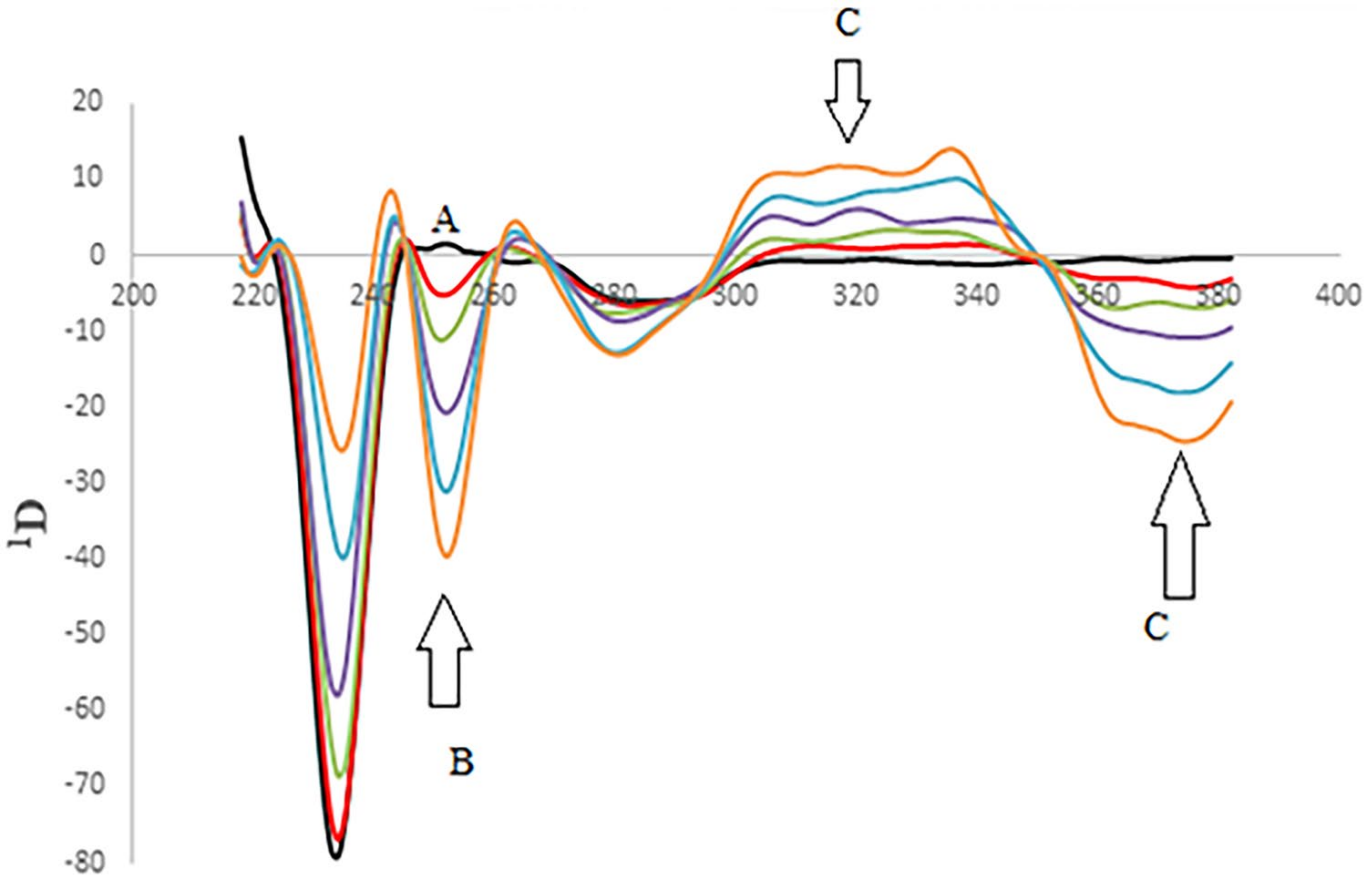
First derivative synchronous fluorescence spectra in spiked plasma where: (**A**) Blank Plasma. (**B**) RMD (**C**) FVP

To test the applicability of the proposed method in different matrices; synthetic mixtures and spiked plasma samples containing the two drugs were evaluated. Different synthetic mixtures with variable ratios were analyzed as in Table 4 and compared also with previous reports to ensure satisfactory results. It was indicated that the mean plasma concentration–time profiles of RMD after intravenous administration are 80.7 ng/mL and 171 ng/mL [28]. For FVP; the concentration after 8 h from the first dose was about 1 µg/mL [29]. Hence, the proposed method could detect both drugs within the biological concentration levels (Table 5). Figure 6 shows different synthetic mixtures in spiked human plasma samples with well-resolved spectra. From the results of these applications, it was found that the method offers satisfactory selectivity for simultaneous analysis of both drugs.

**Table 4:** Results for the estimation of RMD and FVP in synthetic mixtures

Parameters	**Proposed method**				**Comparison** **method **[12, 17]	
	**Conc. taken** **(ng/mL)**	**Conc. taken** **(ng/mL)**	**% Found**^**a**^		**% Found**^**a**^	
	**RMD**	**FVP**	**RMD**	**FVP**	**RMD**	**FVP**
	100.0	30.0	98.51	100.43	98.74	98.9
	50.0	50.0	100.84	100.96	101.12	101.16
	40.0	80.0	100.49	98.51	99.64	99.61
	30.0	100.0	98.38	101.71		
Mean			99.56	100.40	99.83	99.89
± S.D			1.29	1.37	1.20	1.16
%RSD			1.30	1.36		
%Error			0.65	0.68		
t*			0.29 (2.30)	0.29 (2.30)		
F *			1.15 (19.16)	1.39 (19.16)		

^*^ The tabulated t and F values are respectively at *p* = 0.05 [27]

^a^ each result is average of three determinations

**Table 5:** Results for the determination of RMD and FVP in spiked human plasma

Parameter	**RMD**			**FVP**				
	Amount taken (ng/mL)	Amount found (ng/mL)	% Found*	Amount taken (ng/mL)	Amount found (ng/mL)	% Found*	Amount found (ng/mL)	% Found*
		(at 250.6 nm)			(at 335 nm)		(at 377 nm)	
	20.0	22.983	114.92	40.0	45.109	112.77	44.024	110.06
	40.0	37.899	94.75	60.0	55.814	93.02	55.781	92.97
	60.0	58.56	97.6	80.0	78.819	98.52	79.639	99.55
	80.0	81.323	101.65	100.0	101.803	101.8	101.341	101.34
	100.0	100.152	100.15					
Mean			101.81			101.53		100.98
S.D			7.78			8.20		7.04
% RSD			7.64			8.32		6.97
%Error			3.48			4.16		3.52

^*^N.B. Each result is the average of three separate determinations

## Conclusion

Since both RMD and FVP are important antiviral drugs nowadays, establishing new methods for their determination in different matrices remains a necessity and a challenge for researchers. In this research, we assessed RMD and FVP simultaneously using the sensitive spectrofluorimetric technique. This new methodology permitted their concurrent determination with satisfactory precision and accuracy. The linear ranges were 20.0–100.0 ng ml^−1^ and 40.0–100.0 ng ml^−1^ for RMD and FVP, respectively. Based on our optimization conditions, ethanol was the optimum solvent yielding suitable results for both drugs with green characters. The findings suggest that the new spectrofluorimetric method is appropriate for quantifying RMD and FVP in pharmaceutical dosage forms and spiked plasma samples. The method has different advantages including low detection limit, ease of operation, availability, and simplicity.

## Data Availability

All data analyzed during this study are included in this published article and raw data are available from the corresponding author on reasonable request.
